# Patient with three separate accessory saphenous veins identified while graft preparation: A case report

**DOI:** 10.1186/1749-8090-10-S1-A230

**Published:** 2015-12-16

**Authors:** Ufuk Yetkin, Börteçin Eygi, Ertürk Karaağaç, Köksal Dönmez, Ali Gürbüz

**Affiliations:** 1 1 Department of Cardiovascular Surgery, Katip Celebi University Izmir Ataturk Training and Research Hospital, Izmir, Turkey

## Background/Introduction

Congenital anomalies which do not cause functional or cosmetic problems are usually incidentally identified at diagnostic researches or surgical explorations.

## Aims/Objectives

Our case was 54 year-old male patient. He had cerebral ischemic attack a year ago and amaurosis fugax 2 months ago, in his medical history.

## Method

Patient's coronary angiography and selective arcus aortography revealed 50% stenosis at left main coronary artery and serious three-vessel disease. Cardiology and Cardiovascular surgery council decided that ulcerous lesion at left carotid artery has priority. Patient underwent coronary revascularization three weeks after left carotid endarterectomy.

## Results

Under general anesthesia, median sternotomy was performed. LIMA graft preparation and great saphenous vein preparation from right lower extremity were maintained together. Three separate accessory saphenous veins were visualized below knee level. The medial one was thin but other two had optimal diameter. For reducing incision length and surgical wound size, both accessory saphenous veins were prepared by controlled saline infusion and collaterals were ligated. Three coronary arteries were successfully revascularized. The patient recovered uneventfully. Patient is still followed up by our outpatient clinic.

## Discussion/Conclusion

As in our case, latent congenital anomalies are usually incidentally identified at surgical explorations. Sometimes this situation may be altered for patient's advantage and be beneficial for patient even by reducing tissue damage.

## Consent

Written informed consent was obtained from the patient for publication of this Case report and any accompanying images. A copy of the written consent is available for review by the Editor-in-Chief of this journal

